# Properties of mixture of hemp bast and softwood pulp for filter paper manufacture

**DOI:** 10.1016/j.heliyon.2024.e25353

**Published:** 2024-02-01

**Authors:** Piyawan Yimlamai, Korawit Chitbanyong, Kapphapaphim Wanitpinyo, Buapan Puangsin, Kawinthida Nanta, Somwang Khantayanuwong, Sawitree Pisutpiched, Tanapon Chaisan, Binqi Fei, Salim Hiziroglu

**Affiliations:** a Department of Forest Products, Faculty of Forestry, Kasetsart University, Bangkok, 10900, Thailand; b Department of Agronomy, Faculty of Agriculture, Kasetsart University, Bangkok, 10900, Thailand; c Key Laboratory for Forest Resources Conservation and Utilization in the Southwest Mountains of China, Ministry of Education, Southwest Forestry University, Kunming, 650224, China; d Department of Natural Resource Ecology and Management, Oklahoma State University, Stillwater, OK, 74088, USA

**Keywords:** Hemp bast fiber, *Cannabis sativa* L., Soda pulping, Chemical composition, Papermaking

## Abstract

The objective of this study was to investigate the morphological and chemical properties of hemp bast RPF1 variety fiber to be used as a potential raw material for filter paper production.

Experimental handsheet samples with basis weight of 20 g/m^2^ were manufactured using mixture of hemp and softwood pulp at various beating levels. The average fiber length and width of hemp bast fiber were determined as 5.76 mm and 32.53 μm, respectively. It was also found that the hemp bast fiber had rigid thick cell wall with small size of lumen. The overall chemical properties of hemp bast were similar to those fibers from other bast sources as well as softwood fibers. It seems that hemp bast was easily pulped under various soda process conditions yielding pulp ranging from 51.36 % to 52.56 % and Kappa numbers ranging from 2.89 to 8.18. Based on the findings in this study hemp bast fiber could be considered as a potential to manufacture filter paper with accepted characteristics.

## Highlights

• Hemp bast has been investigated in terms of fiber morphology, chemical composition, soda pulping and handsheet properties for the first time in Thailand.
• Hemp bast pulp had high soda pulp yield and low Kappa number.
• Filter paper from mixed hemp bast and softwood pulp had good physical properties with accepted air permeability and surface roughness.
• Hemp bast can be used as alternative raw materials for filter paper manufacture.

## Graphical abstract

**Figure:**
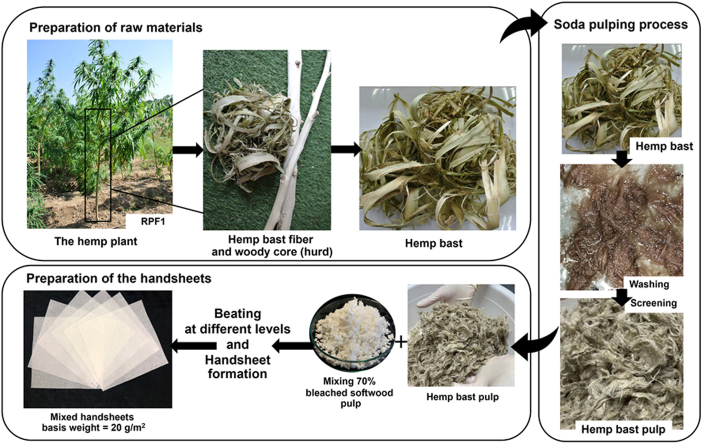

## 1 Introduction

Increased use of the micro-and nano-sized plastics for many applications such as plastic grocery bags is an important concern regarding their adverse impact on the environment. Polyethylene is globally one of the most widely used plastics for many applications including food packaging, i.e., plastic wrap, food bags and beverage caps. Various reports found that plastic bags are causing serious health damage to humans, animals and environmental pollution [1]. One of the most common uses of plastic bags is filter paper. It is widely used for teabags and it has been reported that such teabags release certain amount of micro-and nanoparticles into tea [[2], [3], [4]]. It is a well-accepted fact that urgent attention is required for using biobased resources including plant fiber feedstock as an alternative raw material with no negative influence on the environment.

Global trends toward sustainable development have brought to light natural, renewable, biodegradable raw materials, including plant fibers [5]. Plant fibers play an important role in the current paper manufacture and can be classified according to their origin. They are categorized by the location of the fiber, namely stem, leaf, seed, and bast [6]. Bast fibers are collected from the phloem or bast surrounding the stem of certain dicotyledonous plants [7,8]. These types of fiber have higher tensile strength than that of other natural fibers, so that they could be ideal to be used for different applications in many industries, including textiles, non-woven, rope and nets, as well as pulp, paper and paper board production [9,10]. In the last few decades, research projects were carried out on utilization of bast fiber [[11], [12], [13]] due to attractive features of the bast fibers, namely their low cost, light weight and high specific modulus. Consequently, many scientists tried to use this resource as an alternative synthetic fiber as a reinforcement material in the composite panel manufacture [7,14].

Hemp or industrial hemp (*Cannabis sativa* L.) is a plant in the family Cannabaceae cultivated for its bast fiber or edible seeds. Hemp with a fast growth rate and a high cellulose content ranging from 70 % to 74 %, has highly fiber quality for various end-use products [15,16]. Hemp plant stalks are composed of two fiber sources, an inner hurd layer and an outer bast layer, in which each fiber source has the potential to serve multiple applications [17]. Past researches have reported the use of various parts of industrial hemp ranging from core to the bast fibers in paper manufacturing [[18], [19], [20], [21]]. Industrial hemp has emerged as a highly successful commercial crop due to its attractive carbon-sequestering, higher biomass production, and various end-use products. The hemp fiber also has several advantages such as ease of preparation, uniform material quality, easy liquid penetration, cooking, and high-quality pulping performance [16,20]. The result of previously studied stated that the common pulping process for non-wood is the soda process, such as bagasse, rice straw, hemp, kenaf and flax. Owing to non-wood generally contain less lignin than wood resulting in easily separating lignin. In addition, soda pulping process is relatively simple and requires low capital investment. It is a chemical process that is environmentally friendly and makes strengthened fibers for papermaking [[22], [23], [24]].

Fiber production from hemp has been conducted over many centuries [16,25], Although hemp is illegal in many countries, including Thailand due to its similarities to cannabis which contains high content of Delta-9-Tetrahydrocannabinol (THC) this prohibition was relieved for industrial hemp after being its correct classification. Since Canada, the EU, and some of the other countries made it legal to grow industrial hemp, its cultivation has increased significantly [25]. Currently it is now legalized and promoted by the policy of the Ministry of Public Health in Thailand. This policy not only that will be used for public health but also for boosting the local grassroots economy of Thailand [26]. It is expected that such resource will be one of alternative fibers as a raw material for pulp and paper industry in Thailand and other countries.

To our knowledge, relatively limited information exists related to hemp bast as a pulping raw material. Thus, the main objective of this study was to investigate and quantify the bast fiber properties related to pulp and papermaking. The morphology, chemical composition, and soda pulp properties bast fiber were also determined within the scope of this work. Hemp bast pulp were mixed with softwood pulps in a ratio of 30–70 (w/w) and to improve its strength by beating with different levels. The properties of mixed hemp/softwood of handsheets, with a basis weight of 20 g/m^2^, were determined for the purpose of lightweight filter paper application. Surface morphology of handsheets were also evaluated by the images of scanning electron microscope to have a better understanding of properties of such type of fiber.

## 2 Materials and method

### 2.1 Preparation of raw material

**Fig. 1:**
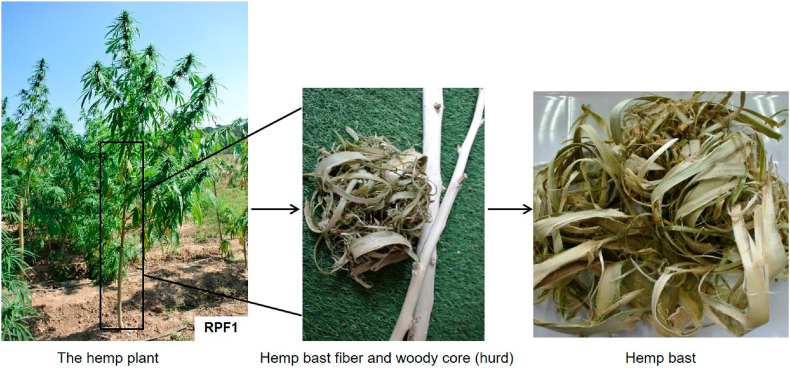
RPF1 hemp plant and hemp bast fiber samples.

The stalks of 3 to 4-month-old RPF1 hemp (Cannabis sativa L.) variety were collected from a plantation located in Pang Mu Sub-district, Mueang Mae Hong Son district, Mae Hong Son, Thailand as illustrated in Fig. 1. Bark and core of hemp plant were manually separated before the moisture content of bast fiber samples were determined according to TAPPI T 258 om-11. Commercial softwood pulps were provided by Thai Paper Co., Ltd. Bangkok, Thailand. Laboratory grade sodium hydroxide (NaOH), glacial acetic (CH_3_COOH), hydrogen peroxide (H_2_O_2_), and all other chemicals and solvents were purchased from Merck Co., Ltd. Bangkok, Thailand without any purification.

### 2.2 Fiber morphology of the samples

Peeled pieces of hemp bast were macerated with a mixture of glacial acetic acid and 30 % hydrogen peroxide in ratio of 1:1 at a temperature of 75 °C for 48 h, according to the Franklin's method (Franklin 1945). After the completion of maceration process, the samples were rinsed with distilled water, before being disintegrated and stained with 1 % safranin. This was followed by an analysis of fiber morphology using a light microscope, BX50; Olympus, Tokyo, Japan. The fiber length, fiber width, lumen width, and cell wall thickness was measured for 100 stained fibers. Using these measurements, the slenderness ratio, fiber length/fiber width, Runkel ratio, 2 × cell wall thickness/lumen width, and ﬂexibility, lumen width/fiber width, were then calculated.

### 2.3 Determination of chemical composition of the samples

The hemp bast samples were cut into a small pieces and powdered using a laboratory mill, Thomas-WILEY Model 4; Arthur H. Thomas Company, Philadelphia, PA, USA. The air-dried hemp bast powder was then screened through a 40-mesh and retained on a 60-mesh screen before the chemical analysis were carried out. Holocellulose content was measured using the extractive-free wood method described previously [27]. Other chemical analyses were carried out based on the alpha cellulose (TAPPI T 203 cm-09), acid-insoluble lignin (TAPPI T 222 om-11), ash (TAPPI T 211 om-12) and alcohol-benzene (TAPPI T204 cm-07) extractives.

### 2.4 Soda pulping of the samples

The soda pulping was done using a laboratory rotating batch reactor with a digester of 7-L capacity, SEW-Eurodrive, Bruchsal, Germany. Three hemp bast samples weighing 300 g under oven-dry conditions were individually subjected to pulping using 16 %, 18 %, 20 %, 22 % and 24 % NaOH, based on the mass of solute per volume of solvent. The liquor to hemp bast fiber ratio was 5:1 and kept at a maximum temperature of 165 °C with the time required to reach maximum temperature being 60 min. The temperature was maintained at this maximum value for 60 min for sample cooking as indicated in Table 1. The obtained brown stock was washed and disintegrated before being screened through a plate with an opening of 0.15 mm, after which the overall pulp yield and reject content percentage was calculated. The kappa number of pulp was analyzed according to TAPPI T236 om-06. The hemp bast pulp samples, after being screened, were gathered and stored in a refrigerator to be used in the production of handsheets.

**Table 1:** Cooking conditions of soda pulp samples.

Parameter	Condition
Liquor to wood ratio	5:1
Active alkali, as NaOH (%)	16, 18, 20, 22 and 24 %
Temperature (°C)	165 °C
Time to temperature (min)	60 min
Time at maximum temperature (min)	60 min

### 2.5 Preparation of the handsheets

**Fig. 2:**
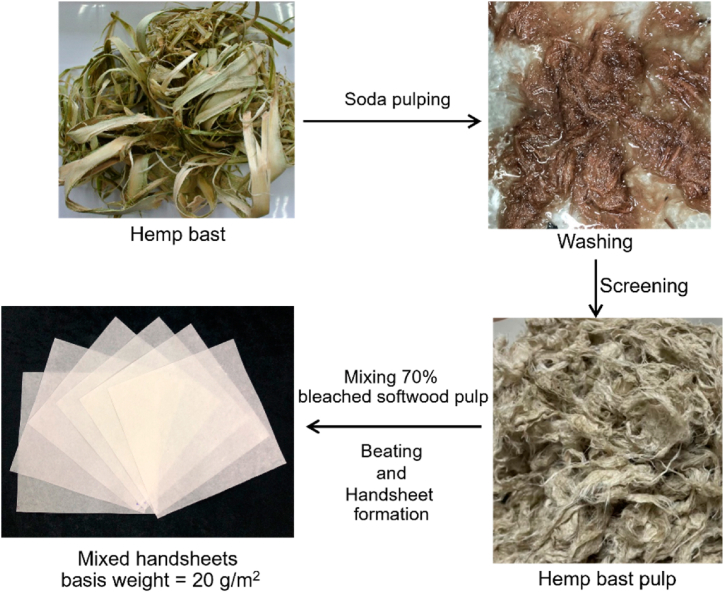
Preparation of hemp bast handsheet samples.

The handsheets with mixture of 70 % of bleached softwood pulp as reinforcement and 30 % hemp bast pulp were made as depicted in Fig. 2. The mixed pulps were refined in a PFI mill, Kumagai Riki Kogyo Co. Ltd., Tokyo, Japan at different beating levels of 0, 10,000 and 12,000 revolutions. The drainability of the samples was assessed using the TAPPI T227 om-09 method. Handsheets with a grammage of 20 g/m^2^ were prepared according to TAPPI Test Method T205 sp-95. All of the samples were dried and conditioned at 23 ± 1 °C and 50 ± 2 % relative humidity (RH) for a week. The physical properties of the samples, encompassing apparent density (measured by TAPPI T220 sp-10) and thickness (determined through TAPPI T411 om-08) using a precision micrometer from Laurentzen and Wettress in Stockholm, Sweden, were determined. Surface roughness of the specimens was additionally measured following the guidelines of the ISO 5636-3 standard, utilizing an automatic Bendtsen apparatus from Frank-PTI Quality Testing Instrument in Vorchdorf, Austria.

The air permeability of handsheet samples was measured through the Gurley method, in accordance with TAPPI T460 om-11, employing an oil-sealed-type densometer model no. 4110 N from Gurley Precision Instruments in New York, USA. Optical properties such as brightness and opacity were gauged using a reflectometer (Model-3; Kumagai Riki Kogyo Co. Ltd., Tokyo, Japan) in accordance with TAPPI T452 om-08 for brightness and TAPPI T452 om-11 for opacity, respectively.

For each type of sample, ten handsheets were utilized to assess their mechanical properties, specifically tensile strength (following TAPPI T494 om-06), tearing strength (in accordance with TAPPI T414 om-04), bursting strength (according to TAPPI T403 om-10), and folding endurance (using TAPPI T511 om-08). These evaluations were conducted using a tensile tester (EJA-series; Thwing-Albert Instrument Co. Ltd., West Berlin, USA), tearing resistance tester (Thwing-Albert Instrument Co. Ltd., West Berlin, USA), bursting strength tester (Laurentzen and Wettress, Stockholm, Sweden), and folding endurance tester (Kumagai Riki Kogyo Co. Ltd., Tokyo, Japan), respectively.

### 2.6 Microscopic evaluation of the handsheet samples

Handsheet surface morphology was observed under a scanning electron microscope (SEM), SU3900; Hitachi, Tokyo, Japan. The unbeaten and beaten pulp handsheet were also analyzed on a scanning electron microscope. Handsheet samples were photographed at 100x and 300x magnification.

## 3 Results and discussion

### 3.1 Fiber morphology of the samples

**Fig. 3:**
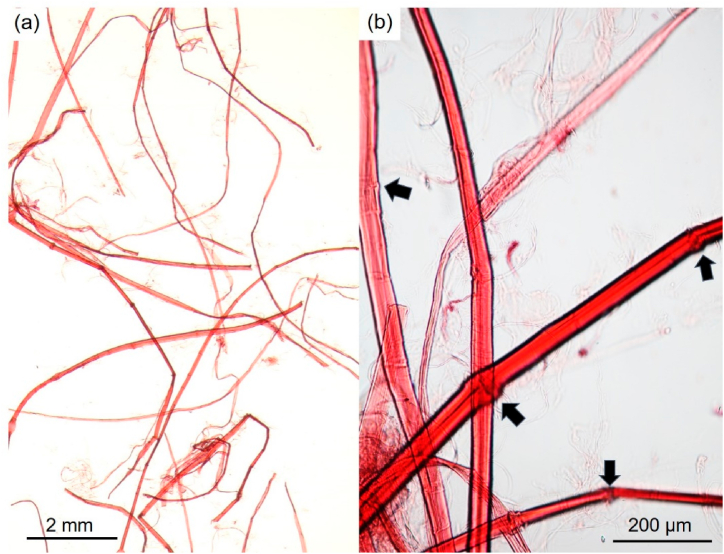
Microphotographs of hemp bast fiber: (a) overview of hemp bast fiber (at 40x magnification) and (b) zoomed-in snapshot of a region depicting transverse node along a degummed hemp bast fiber (at 400× magnification).

The fiber morphology plays an important role in determining the overall strength properties of a paper depending on not only structural properties of fiber network but also strength of individual fibers. The microphotographs of hemp bast fiber are shown in Fig. 3 and properties of hemp bast fiber are listed in Table 2. The morphological analysis showed the long and round fiber as illustrated in Fig. 3a identifies some nodes as can be seen in Fig. 3b which is very typical and common in the case of hemp and other bast fiber. Bast fibers possess transverse nodes and fissures in cross-sectional and longitudinal directions. The existence of the nodes and the fissures in the bast fibers could degrade the fiber mechanical properties [28].

**Table 2:** Comparison of morphological properties of hemp bast, other type of bast fibers and softwoods.

Properties	Hemp bast	Coi bark^a^	Kenaf^b^	Softwoods^a^
Fiber length *(L)*, mm	5.76 ± 0.32	12.10 ± 0.28	2.55 ± 0.60	3.27 ± 0.14
Fiber width *(D)*, μm	32.53 ± 1.62	23.24 ± 0.68	22.35 ± 1.20	37.12 ± 1.41
Lumen width *(d)*, μm	9.38 ± 0.47	6.40 ± 0.25	5.23 ± 0.50	13.93 ± 0.99
Wall thickness *(w)*, μm	11.57 ± 0.71	8.42 ± 0.32	11.90 ± 0.50	11.59 ± 0.72
Slenderness ratio *(L/D)*	192.39 ± 13.17	530.45 ± 18.32	114.23 ± 28.18	89.29 ± 3.16
Runkel ratio *(2w/d)*	2.65 ± 0.14	2.75 ± 0.17	0.88 ± 0.10	1.99 ± 0.24
Flexibility ratio *(d/D) x100*	29.89 ± 10.80	28.00 ± 1.00	53.24 ± 3.60	38.00 ± 3.00

Note: ^a^ [29]; and ^b^ [30].

The average length and width of hemp bast fiber were found as 5.76 mm and 32 μm, respectively as displayed in Table 2 which was identical to within the range of 5–55 mm that of reported in previous studies [16,25,31]. The average fiber length of hemp bast is also comparable to other bast fiber source such as kenaf (*Hibiscus cannabinus*) (2–11 mm), jute (*Corchorus olitorius*)(1.5–5 mm), flax (*Linum usitatissimum*) (<5 mm), and ramie (*Boehmeria nivea*) (<5 mm) determined in various past works [[32], [33], [34], [35]].

Fiber length of hemp bast fiber was found shorter than that of Coi bark (*Streblus aspen* Lour.) which another bast fiber source in Thailand but longer than that of any softwood species.

The hemp bast fiber width, lumen width, and wall thickness was measured at 32.53 μm, 9.38 μm, and 11.57 μm, respectively. This implies that the hemp bast fiber has a high cell wall thickness with a small lumen. Such characteristics of the hemp bast fiber have been previously reported as well [36,37].

Hemp bast fiber had a very good slenderness ratio (>60) which is higher than that of kenaf and softwoods creating an excellent fiber bonding and increasing strength of the paper [38]. However, hemp bast fiber is still rigid having a flexibility ratio less than 30 and poor Runkel ratio (Runkel ratio >1) indicating that the fibers do not easily collapse when compared to the fibers of kenaf (Runkel ratio <1). As such, a higher beating level might be required to develop the required strength of the paper. However, the observed low flexibility is would negatively affect the fiber strength properties [38].

### 3.2 Chemical composition of the samples

As indicated by the chemical composition of hemp bast in Table 3. Hemp bast exhibited a greater holocellulose content in comparison to kenaf and mulberry. However, the lignin content and extractive in the hemp bast was lower than kenaf. The ash content measured for the hemp bast was higher than kenaf and mulberry but lower than Coi bark. These results exhibit the potential of hemp bast as a raw material in pulp production. The alpha cellulose content in hemp bast correlates directly with pulp yield. The holocellulose and alpha cellulose in the hemp bast were 75.93 and 51.25 % respectively, which have the potential for use as pulp material in pulp production [39]. A previous study indicated the mean cellulose and hemicellulose content of hemp bast were 64.8 % and 7.7 %, respectively [40]. Considering the amount of lignin in hemp bast relative to general hemp bast lignin was 3.3–5.5 % [40,41]. Hemp bast ash content was 4.82 %, which is higher than that in softwoods and hardwoods [42,43], and it can also be referred to as the silica content. The high content of ash can cause problems in pulping process, such as refining and recovery system [38,44,45]. There were about 1.83 % ethanol-benzene extractives and other extractives in the hemp bast. These extractives can include various organic compounds found within the hemp bast, such as pectin, fats, oils, pigment and other soluble substances [21].

**Table 3:** Comparison of chemical composition of hemp bast with other bast fiber sources.

Chemical Composition	Content (% Oven-dry Weight of Raw Material)			
	Hemp bast	Coi^a^	Mulberry^a^	Kenaf^b^
Holocellulose	75.93 ± 0.28	79.62 ± 0.17	71.03	72.31
Alpha cellulose	51.25 ± 0.71	65.52 ± 0.24	62.14	48.20
Hemicellulose	24.68 ± 0.96	14.78 ± 0.22	8.11	19.05
Lignin	9.73 ± 0.57	6.02 ± 0.21	–	16.27
Ash	4.82 ± 0.08	8.45 ± 0.06	4.30	2.87
Extractives	1.83 ± 0.06	6.29 ± 0.63	4.11	3.84

Note: ^a^ [29]; and ^b^ [30].

### 3.3 Soda pulping

The active alkali and pulp properties of hemp bast, including pulp yield, Kappa number and rejects are presented in Table 4. It can be observed that the sample pulp yield and Kappa numbers decreased from 52.56 % to 51.36 % and from 8.18 to 2.89, respectively, with an increase in the concentration of NaOH from 16 % to 24 %. It is known that Kappa number indicates the residual lignin in pulp and the degree of delignification achieved during pulping process. The approximately residual lignin content of the samples slightly decreased from 1.27 % to 0.45 % (calculated as Lignin content = Kappa Number x 0.155) as the concentration of NaOH increased from 16 % to 24 %. This decline in lignin content can be attributed to the solubilization of lignin during the chemical treatment and may be a contributing factor to the reduction in yield [46]. The Kappa number hemp bast was lower than that of kenaf (29.4) [47] and similar to that of jute (10.2) [48]. Previous studies have shown that the soda pulping of industrial hemp bast had higher yield (96.5 %) and lower Kappa number values (11.5) [36,49]. It could be concluded that soda pulping of hemp bast provided mainly average yield pulp as compared to that of soda pulp from kenaf [47] and mulberry [50]. In this study, the optimal concentration of NaOH was found as 24 % for producing hemp bast pulp.

**Table 4:** Pulp yield, reject and kappa number of hemp bast pulp resulted by various level of active alkali in soda process.

Active alkali (%)	Pulp yield (%)	Reject (%)	Kappa number
16	52.56 ± 1.39	1.72 ± 0.57	8.18 ± 1.33
18	52.35 ± 1.38	1.93 ± 0.23	6.51 ± 0.41
20	53.57 ± 1.44	1.54 ± 0.11	4.33 ± 0.06
22	51.55 ± 0.64	1.43 ± 0.08	3.46 ± 0.25
24	51.36 ± 0.74	1.65 ± 0.20	2.89 ± 0.08

### 3.4 Microscopic observations of the sheet sample of mixed hemp/softwood pulp

**Fig. 4:**
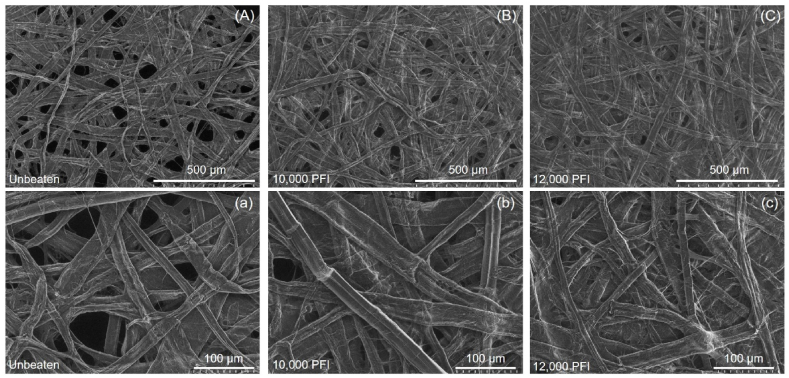
Hemp bast surface SEM micrographs (basis weight = 20 g/m2) for unbeaten handsheets; A = 100x, a = 300x, Beaten at 10,000 PFI revolutions; B = 100x, b = 300x, and Beaten at 12,000 PFI revolutions; C = 100x, c = 300x.

Micrographs of the surface morphology of mixtures 30:70 (w/w) of hemp bast and softwood pulp handsheets before and after beating process take on an SEM are depicted in Fig. 4. Changes in pulp and handsheet characteristics as a result of beating can be observed in the SEM micrographs. Unbeaten pulp handsheets had a high porosity, were bulky, and lower bonding ability with poor formation (Fig. 4A, a). This was in contrast to handsheets produced with beaten pulp which were denser and smoother as shown in Fig. 4B–C, b-c. As mentioned earlier, this observation is also in correspondence with the fiber morphology. The hemp bast were very long in length and did not collapse well due to thick cell wall. This implies that such fibers with small rigid lumen anatomy can result in voids between fibers when compared to the other pulp fibers [51].

### 3.5 Handsheet samples and testing

The furnish of pulp used in making handsheet was a blend with hemp bast and softwood pulp in a ratio of 30:70. The results and standard deviations of the physical and mechanical properties of the samples are displayed in Table 5, Table 6, respectively.

**Table 5:** Change in the physical properties of handsheet at different beating revolutions.

Paper properties	Beating revolutions by PFI mill		
	0	10,000	12,000
Freeness (ml, CSF)	689 ± 4.58	360 ± 1.67	307 ± 6.67
Thickness (mm)	0.06 ± 0.02	0.05 ± 0.01	0.05 ± 0.01
Apparent density (g/cm^3^)	0.29 ± 0.01	0.34 ± 0.01	0.35 ± 0.01
Brightness (%)	64 ± 0.04	61 ± 0.09	60 ± 0.03
Opacity (%)	95 ± 0.41	92 ± 0.49	92 ± 0.17
Roughness (ml/min)	842 ± 10.9	627 ± 9.37	534 ± 21.14
Air permeance (μm/Pa⋅s)	520.99 ± 22.59	318.28 ± 58.48	253.08 ± 38.93

**Table 6:** Change in the mechanical properties of handsheet at different beating revolutions.

Paper properties	Beating revolutions by PFI mill		
	0	10,000	12,000
Dry tensile index (Nm/g)	6.70 ± 0.45	45.46 ± 1.39	41.27 ± 1.67
Wet tensile index (Nm/g)	0.77 ± 0.31	1.00 ± 0.54	0.73 ± 0.22
Tear index (mN.m^2^/g)	14.33 ± 0.36	19.22 ± 1.06	15.85 ± 0.55
Burst index (kPa.m^2^/g)	1.16 ± 0.07	2.49 ± 0.20	2.62 ± 0.134
Folding endurance (double folds)	N/A	1911 ± 199	2212 ± 202

The results indicated that the initial freeness of mixture pulps was higher than that of hardwood and softwood pulp [51]. Beating response, freeness drops due to PFI revolutions which can lead to fibrillation and disruption of fibers, resulting in increased bonding surface area and collapsibility upon beating. The handsheets produced through the beating process displayed increased density and smoothness in contrast to those made from unbeaten pulp. The thickness levels of handsheets, before and after beating process were 0.05–0.06 mm, respectively and handsheets formed from beaten pulp also had higher apparent density of 0.34–0.35 g/cm^3^.

The brightness of handsheets significantly decreased with increasing apparent density of the samples. This observation was likely due to a higher apparent density or fewer air voids, which reduced the refraction and scattering of light traveling through the structure. Beating of pulp fibers resulted in denser handsheets with a lesser number of air voids in their structure, as shown in Fig. 4. Previously, Khantayanuwong et al. [52] had demonstrated that the handsheet brightness increased while the apparent density decreased, which is in close correspondence to a theory stating that the handsheet brightness is a result of the amount of diffusely reflected light caused by refraction and scattering. The difference in the refractive index of transparent cellulose fiber and air voids in the handsheet structure affected both the refraction and scattering of light traveling throughout the structure [53].

Surface roughness and air permeability of paper are two important structural features playing significant role on their applications [54]. Samples made from the beaten pulp had smooth surface comparable to those made with unbeaten pulp increasing due to fibrillation and network. The air permeability values of handsheet were 253.08–520.99 μm/Pa.s. which had lower than filter paper from jute [10]. These results possibly indicate that handsheet from mixed hemp/softwood pulp might slow rate of leaching.

The mechanical properties of unbeaten and beaten handsheets are presented in Table 6. The dry tensile index, burst index and folding endurance of handsheet increased with increasing beating level. This is probably due to the development of internal delamination and fibrillation of pulp fibers, which resulted in an increased bonding ability and collapsibility through the beating process [51,55]. The dry tensile strength of beaten handsheet are in the range of 41.27–45.46 Nm/g. On the contrary, wet tensile strength of all handsheet are in the range of 0.73–1.00 Nm/g, which lower than that of filter paper from jute [10]. As a result, beating level did not affect wet tensile properties of the samples due to the handsheet structural consisting of pure cellulose, thus, the weak wet tensile strength owing to the abundance of accessible hydroxyl groups on its surface caused degradation of the structure. The tear index values of the samples rapidly increased with increasing beating level from 0 to 10,000 revolutions and slowly decreased with increasing beating level from 10,000 to 12,000 revolutions. Intensity increase, changes in fiber length, width as well as coarseness of the samples due to beating decreased proportionally that might have negative effects on paper strength [56].

In this study, filter paper with a basis weight in the range of 20–30 g/m^2^ is considered lightweight and is used for a variety of filtration and separation applications. Here are some common uses for filter paper in this basis weight range, such as those used in tea bags, coffee filters, air and gas filtration, laboratory filtration, or industrial applications, may have distinct sets of standard properties and characteristics. For example, in the context of tea filter paper production, the dry tensile strength (45.46 Nm/g), wet tensile strength (1.00 Nm/g), and air permeability (318.28 μm/Pa.s.) of the beaten handsheets at 10,000 revolutions indicated that the dry tensile strength exceeded the Chinese standard values (15–20 Nm/g). Hence, the lower wet tensile strength and air permeability of the beaten handsheets at 10,000 revolutions, falling below the Chinese standard values (3–4 Nm/g and 500–1690 μm/Pa.s, respectively). This has prompted concerns about the reduced wet strength and the heightened presence of voids within the filter paper [10].

However, those handsheets made from mixed hemp/softwood pulp need to modify to strong enough to be soaked in water. Several studies have suggested various technologies and processes for cellulose modification [[57], [58], [59]]. Therefore, according to the above results, the data presented revealed that it would be feasible to produce filter paper from mixed hemp/softwood pulp even though modification is needed to improve strength properties.

## 4 Conclusions

The fiber morphological, chemical, and soda pulp properties of hemp bast were assessed to determine their suitability as raw materials for the production of filter paper. The findings suggest that hemp bast fibers are longer than those of kenaf and softwoods fibers. The chemical composition of hemp bast revealed a higher alpha cellulose content and lower lignin content, making it a suitable material for papermaking purposes. After the soda pulping process, hemp bast demonstrated a high pulp yield and a low Kappa number, with an optimal NaOH concentration of 24 %. In the preparation of handsheets, hemp bast pulp was blended with softwood pulp in a ratio of 30:70 (w/w). The basis weight of the resulting handsheets was adjusted to a lightweight value, and various beating times were employed. The results indicated that the strength properties of the handsheets enhanced with prolonged beating time, particularly at 10,000 revolutions. It seems that hemp bast has the potential to use as an alternative raw material for the production of filter paper. However, the application of lightweight filter paper in liquid filtration serves the purpose of enhancing wet strength and increasing voids within the filter, thereby improving its filtration performance.

## Data availability statement

Data included in article/supp. Material/referenced in article.

## CRediT authorship contribution statement

**Piyawan Yimlamai:** Writing – original draft, Methodology, Data curation. **Korawit Chitbanyong:** Methodology, Data curation. **Kapphapaphim Wanitpinyo:** Methodology, Data curation. **Buapan Puangsin:** Writing – review & editing, Visualization, Resources, Funding acquisition, Formal analysis, Data curation, Conceptualization. **Kawinthida Nanta:** Methodology. **Somwang Khantayanuwong:** Writing – review & editing. **Sawitree Pisutpiched:** Writing – review & editing. **Tanapon Chaisan:** Funding acquisition. **Binqi Fei:** Methodology. **Salim Hiziroglu:** Writing – review & editing.

## Declaration of competing interest

The authors declare the following financial interests/personal relationships which may be considered as potential competing interests:Buapan Puangsin reports financial support was provided by 10.13039/501100005621Kasetsart University Research and Development Institute (KURDI) (FF(KU)10.64).
